# Initial Report of a Phase I Study of LY2510924, Idarubicin, and Cytarabine in Relapsed/Refractory Acute Myeloid Leukemia

**DOI:** 10.3389/fonc.2018.00369

**Published:** 2018-09-24

**Authors:** Prajwal Boddu, Gautam Borthakur, Mythili Koneru, Xuelin Huang, Kiran Naqvi, William Wierda, Prithviraj Bose, Elias Jabbour, Zeev Estrov, Jan Burger, Yesid Alvarado, April Deshmukh, Ami Patel, Antonio Cavazos, Lina Han, Jorge E. Cortes, Hagop Kantarjian, Michael Andreeff, Marina Konopleva

**Affiliations:** ^1^Department of Leukemia, University of Texas at MD Anderson Cancer Center, Houston, TX, United States; ^2^Eli Lilly and Company, Indianapolis, IN, United States

**Keywords:** LY2510924, idarubicin, cytarabine, relapsed refractory, acute myeloid leukemia, CXC4

## Abstract

**Background:** The CXCR4/SDF-1α axis plays a vital role in the retention of stem cells within the bone marrow and downstream activation of cell survival signaling pathways. LY2510924, a second generation CXCR4, showed significant anti-leukemia activity in a murine AML model.

**Methods:** We conducted a phase I study to determine the safety and toxicity of LY2510924, idarubicin and cytarabine (IA) combination therapy in relapsed/refractory (R/R) AML. Eligible patients were 18–70 years of age receiving up to salvage 3 therapy. A peripheral blood absolute blast count of < 20,000/μL was required for inclusion. LY2510924 was administered daily for 7 days followed by IA from day 8. Two dose escalation levels (10 and 20 mg) were evaluated, with a plan to enroll up to 12 patients in the phase I portion.

**Results:** The median age of the enrolled patients (*n* = 11) was 55 years (range, 19–70). Median number of prior therapies was 1 (1–3). Six and five patients were treated at dose-levels “0” (10 mg) and “1” (20 mg), respectively. Only one patient experiencing a dose limiting toxicity (grade 3 rash and myelosuppression). Three and one complete responses were observed at dose-levels “0” and “1,” respectively; the overall response rate (ORR) was 36% (4 of 11 patients). A ≥ 50% decrease in CXCR4 mean fluorescence intensity was observed in 4 of 9 patients by flow cytometry, indicating incomplete suppression of CXCR4-receptor occupancy.

**Conclusions:** The combination of LY2510924 with IA is safe in R/R AML. Dose-escalation to a 30 mg LY2510924 dose is planned to achieve complete blockade of CXCR4 receptor occupancy, followed by expansion phase at the recommended phase 2 dose-level.

## Introduction

The stromal microenvironment plays a role in hematopoietic stem cell growth and is protective to the leukemic stem cells in the bone marrow (1). The bone marrow stromal cells activate multiple signaling pathways in leukemic cells which influence their proliferation and survival (2). More recently, chemokines or chemotactic cytokines, in particular stromal derived factor 1 alfa (SDF-1α), have been demonstrated to play a crucial role in the maintenance and maturation of the hematopoietic compartment and in the regulation of hematopoiesis.

SDF-1α (also known as CXCL12) mediates its functions through its receptor, chemokine (C-X-C motif) receptor 4 (CXCR4) which is a transmembrane G-protein–coupled receptor (3). The CXCR4/SDF-1α axis is involved in the migration of hematopoietic stem cells (HSCs), and both factors are required for normal murine fetal development (4). Among the downstream transduction pathways activated by CXCR4/SDF-1α interaction are the PI3K/Akt and Ras/Raf/MAPK cascades, two important pathways involved in cell proliferation and survival (5, 6). There is evidence supporting the role of the SDF-1α/CXCR4 in tumor growth and metastasis (7, 8). Studies suggest that interruption of the CXCR4/SDF-1α signaling axis results in peripheral blood migration of the hematopoietic stem cell progenitors from the bone marrow (9–13). Blockade of the CXCR4 and SDF-1α axis have resulted in antitumor efficacy in a variety of preclinical models (8, 14–18).

LY2510924 is a selective CXCR4 antagonist that inhibits SDF-1α binding to CXCR4 (16). LY2510924 shows antitumor activities in a variety of solid tumor xenograft models including non-Hodgkin's lymphoma, non-small cell lung cancer, renal cell carcinoma (RCC), and colorectal cancer. In an experimental breast cancer and lung metastasis xenograft model, LY2510924 inhibited the metastasis of tumor cells to the lung and their continuous growth in the lung (16). In a phase I trial, the maximum tolerated dose (MTD) of LY2510924 was determined to be 20 mg administered subcutaneously (SC) once daily (19). There was a dose dependent response in CD34^+^ cell mobilization between 1 mg and 10 mg, with little additional response with higher doses of 20 or 30 mg. The promising pre-clinical results with LY2510924 have however not translated well in the solid tumor combination therapy trials. In a recently published randomized phase II study, LY2510924 (at 20 mg SC) was added to standard of care chemotherapy for advanced small cell lung cancer (SCLC). The addition of LY2510924 to carboplatin/etoposide while not adding to the combination's efficacy had an acceptable toxicity (20). Two other early phase trials evaluating LY2510924 in combination with durvalumab in solid tumors (NCT02737072) and with sunitinib in RCC (NCT01391130) were terminated due to insufficient efficacy.

Our group and others have tested other CXC4 inhibitors namely AMD3100 (plerixafor) (21, 22), approved by the U.S. Food and Drug Administration (FDA) for stem cell mobilization in multiple myeloma (23), and BL-8040(BTK-140) (24, 25). Both these agents disrupt the SDF-1α/CXCR4 axis and enhance the antileukemic effects of chemotherapy. Based on the encouraging pre-clinical data of CXCR-4 antagonism in AML (26, 27), clinical studies evaluating these agents have been initiated in AML. In a phase 1/2 study of plerixafor combined with mitoxantrone, etoposide, cytarabine (MEC) in relapsed/refractory (R/R) AML, a response rate of 46% was achieved, significantly improved over response rates with MEC alone (28). Our group has reported encouraging responses in relapsed AML patients harboring *FLT3* mutations when plerixafor was combined with sorafenib (29). Another agent undergoing active clinical investigation is BL-8040, a high affinity peptide CXCR4 inhibitor with a prolonged pharmacodynamic efficacy and direct pro-apoptotic activity on AML blasts (24, 25). In a phase 1/2 trial of patients with R/R AML (NCT01838395), patients received 2 days of BL-8040 monotherapy followed by 5 days of BL-8040 and cytarabine combination. The composite complete remission rate achieved during dose escalation (*n* = 22) was 38% (30). Encouraging clinical responses with these CXCR4 antagonists provides a proof of concept for CXCR4 inhibition as a valid therapeutic approach in AML. In an acute myeloid leukemia (AML) model, LY2510924 showed antitumor activity in combination with chemotherapy as well as monotherapy (31). Anti-leukemic activity was equivalent between LY2510924 alone and chemotherapy alone, with the most impressive response observed when LY2510924 was combined with chemotherapy. Based on these findings, CXCR4 antagonists not only having single agent activity but also enhance anti-leukemia effects of cytarabine and doxorubicin in AML.

The mobilization effect on leukemic blasts with plerixafor is transient, and cell counts return to baseline levels within 12 h. Plerixafor has a short half-life *in vivo* and is an incomplete inhibitor of the SDF-1α/CXCR4 axis (22, 28). The rationale for CXCR4 inhibition and the preclinical data with more potent, longer acting 2nd generation CXCR4 antagonist LY2510924 provide basis for the current study with expectations to improve responses and duration of response in AML patients. This phase 1b clinical trial was initiated in patients with R/R AML to evaluate the safety and feasibility of LY2510924 in combination with idarubicin/cytarabine chemotherapy.

## Methods

### Patient selection

This open-label, single-arm, phase 1 study is conducted at The University of Texas MD Anderson Cancer Center (NCT02652871). Patients aged 18–70 years were selected based a histologically or cytologically confirmed diagnosis of AML [except acute promyelocytic leukemia] with R/R disease (refractory to a non-high-dose cytarabine-containing regimen only) receiving their 1st, 2nd, or 3rd salvage irrespective of the genetic abnormality; patients with secondary AML were also included. Clinical laboratory values required a baseline white blood count < 30,000/μL and absolute blasts in peripheral blood (PB) < 20,000/μL. Other eligibility criteria included patient performance status of 0–2 (per Eastern Cooperative Oncology Group), creatinine clearance > 40 mL/min, bilirubin ≤ 2.0 mg/dl and SGOT or SGPT ≤ 3 times the upper limits of normal (ULN), and a normal cardiac ejection fraction. All patients were enrolled onto the study after the approval of the institution's institutional review board and written informed consent obtained before enrollment in accordance with the Declaration of Helsinki.

### Treatment plan

**Figure 1:**
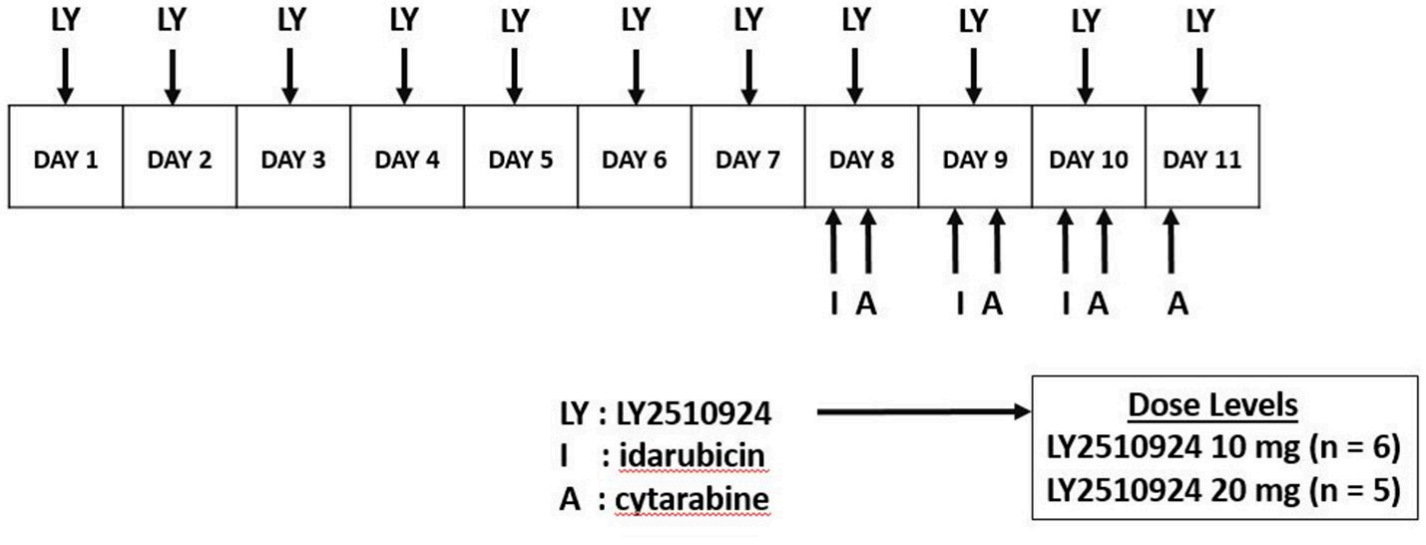
Dosing schedule. LY2510924 was given as monotherapy on days 1–7. Idarubicin and cytarabine chemotherapy was administered starting day 8 along with LY2510924.

LY2510924 was administered daily for 7 days (days 1–7) as monotherapy by SC route. The dose escalation of LY2510924 included the following dose levels: 10 (starting dose), 20, and 30 mg/d. The standard 3+3 algorithm was implemented for dose escalation; 3–6 patients were enrolled on each dose level, with escalation to the next level if dose limiting toxicity (DLT) was encountered in 0 of 3 or 1 of 6 patients. The maximum tolerated dose (MTD) level was defined by the highest dose for which no more than 1 DLT occurred among 6 patients, and would be chosen at the recommended phase 2 dose. If the absolute blast + monocyte count remained < 50,000/μL on days 1–7, chemotherapy was initiated on day 8 consisting of: idarubicin 12 mg/m^2^ intravenous (IV) approximately over 1 h daily × 3 days and cytarabine 1.5 gm/m2 IV approximately over 24 h daily for 4 days; in patients > 60 years of age, idarubicin was given for 2 days and cytarabine for 3 days only (Figure 1). Starting on day 8, LY2510924 would be administered once daily prior to administration of idarubicin and discontinued upon completion of the chemotherapy phase. If, however, counts increased to ≥ 50,000/μL 24 h following the LY2510924 injection during the monotherapy phase, patients could proceed directly to the combination therapy stage provided there were no signs of leukostasis.

Patients who derived a clinical benefit e.g., complete remission (CR), CR with incomplete platelet recovery (CRp), or complete remission without incomplete blood count recovery (CRi) would receive up to four to six additional cycles of LY2510924 in combination with chemotherapy at the consolidation doses. LY2510924 would be administered at the same dose as during induction, concomitant with consolidation therapy consisting of attenuated doses of idarubicin and cytarabine (IA), and discontinued thereafter.

### Treatment evaluation and correlative studies

Pre-treatment evaluation included detailed history, physical examination, CBC with manual differential, comprehensive metabolic panel, lactate dehydrogenase, total bilirubin, AST, and/or ALT, bone marrow aspirate and/or biopsy (examined by H&E and flow cytometric analysis for assessment of leukemic cell numbers), electrocardiography, and echocardiogram. Bone marrow (BM) aspirates were performed at baseline, on day 8 prior to starting IA, and on day 28 for assessment of response; BM assessments were performed after cycle 4 and cycle 6 of treatment during consolidation.

Patients must have received a minimum of one cycle of treatment to be considered eligible for analysis of response. Response to treatment was assessed according to International Working Group standards for AML (32). Overall response rate (ORR) was calculated based on the composite complete remission rates (CR + CRp + CRi) achieved after 1–2 induction cycles.

Correlative studies included the evaluation of CXCR4 and Very Late Antigen-4 integrin (VLA4) molecule expression at baseline and after LY2510924 administration, and mobilization of AML blasts and stem/progenitor cells. CXCR4 receptor occupancy was measured using the CXCR4 antibodies, 12G5 and 1D9, in the peripheral blood (PB) on days 1 and 3, at pre-dose, 4, and 24 h post-LY2510924. The proportion of AML stem/progenitor cells were analyzed based on CD34+38-CD123+ phenotype by flow cytometry (Supplemental Figure 1).

### Statistical considerations

For a subject to be eligible for DLT evaluation, he/she should have received 70% (7 days) of the planned doses of LY2510924 in cycle 1, unless the doses were omitted for DLT defining event. Once the MTD is identified, plan was to expand the trial such that up to 18 total additional patients may be accrued at the MTD.

For categorical variables, frequency tables including percentages will be presented. For continuous variables, descriptive statistics such as median and range were tabulated. Survival endpoints were estimated using the Kaplan-Meier approach. Pharmacodynamic biomarkers were summarized using descriptive statistics.

## Results

### Study population

Eleven patients with R/R AML were treated on this protocol. Table 1 outlines the baseline patient and disease characteristics of the study population. The patient population had a median age of 55 years (range, 19–70). All enrolled patients had experienced treatment failure to at least 1 prior therapy (median: 1, range: 1–3). Eight (72%) of the 11 patients had a first remission (CR1) duration lasting < 12 months. Three (27%) had a prior allogeneic transplant (ASCT) and five (45%) were previously treated with cytarabine based chemotherapy; none had secondary AML. Among the cases analyzed, two (20%) had complex cytogenetics and one (11%) had a *TP53* mutation (Table 1).

**Table 1:** Baseline patient and disease characteristics (*n* = 11).

**Characteristics**		**No./proportion (%); or Median [Range]**
Age in years		55 [19–70]
White blood cell count, K/microL		1.8 [0.7–8.9]
Platelet count, K/microL		28 [3–146]
Peripheral blood blast percentage, %		14 [2–50]
Lactate dehydrogenase, IU/L		461 [338–1172]
Bone marrow blast percentage, %		27 [7–82]
Prior therapies		1 [1–3]
Prior transplant		3 (27)
Secondary AML		0 (0)
Intermediate-dose cytarabine based		5 (45)
Hypomethylating therapy		5 (45)
Cytogenetics	Adverse risk	2/10 (20)
	Diploid	5/10 (50)
	Miscellaneous	2/10 (20)
	Favorable risk	1/10 (10)
*RUNX1*		2/9 (22)
*IDH1/2*		2/9 (22)
*RAS*		1/9 (11)
*NPM1*		1/9 (11)
*DNMT3A*		1/9 (11)
*TP53*		1/9 (11)
*TET2*		1/9 (11)

### Dosing history

Two dosing levels of (a) 10 mg/day of LY2510924 (dose level “0”) and (b) 20 mg/d of LY2510924 (dose level “1”) were explored in the study. A total of 20 cycles were administered to 11 patients; 5 patients received more than one cycle. One patient (in dosing level “1”) required a 2-day dose interruption of LY2510924, on days 3 and 4 of treatment, due to a transient rise in the absolute blast + monocyte count to higher than 50,000/μL, and was subsequently started on attenuated-dosed combination therapy (aged > 60 years) on days 5 and 6 along with resuming LY2510924 after the absolute blast + monocyte count had returned to below 50,000/μL. This patient required to be taken off study as she didn't receive the minimum mandated 70% of LY2510924 dosing, due to being held for elevated absolute blast + monocyte count per protocol.

### Clinical responses

Nine of the 11 patients enrolled onto the study were evaluable for objective response (Table 2). Of the two cases deemed ineligible in this cohort, the first was due to not receiving 70% of LY2510924 dosing following a transient rise in the absolute blast + monocyte count to > 50,000/μL. The second case was in-evaluable due to development/identification of lymphoma during the first cycle of treatment. Neither of these two patients had an objective response of AML at the end of the first cycle. Notably, the patient with lymphoma had a marked PET-CT response with considerable decrease in lymph node size and near resolution of FDG-avidity at the sites involved by lymphoma.

**Table 2:** Disease characteristics, responses, and outcomes of all patients.

**Dosing cohort/accrual number**	**Prior treatment/CR1 duration**	**Salvage status**	**Cytogenetics**	**Mutations**	**Dose limiting toxicities**	**Response**	**Duration of response**	**Reason for withdrawal**	**Comments**
“0”/#1	IA + transplant/12 yrs	1	Diploid	None	Two G3	CRi	1.5 mo	death	Course 2 delayed due to myelosuppression
“0”/#2	7 + 3/1.5 yrs	1	*t*_(8, 21)_	*JAK2*	None	CR, MRD+	3.5 mo+	ASCT	Died post-transplant from MI
“0”/#3	7 + 3/0.5 yrs	1	Diploid	*IDH2*	None	NR	–	NR	Transitioned to IDH inhibitor
“0”/#4	7 + 3, FIA + ASCT + azacitidine/0.25 yrs	2	Complex	*DNMT3A*	None	NR	–	NR	No response to reinduction. Died of progressive disease a month later
“0”/#5	CIA, FLAG-IDA + ASCT, decitabine/0.75 yrs	3	del(9q)	ND	None	NR	–	NR	Died of sepsis 3 months post study withdrawal
“0”/#6	FIA/3 yrs	1	Diploid	*RUNX1*	None	CRi	1 mo+	ASCT	10% blasts after first cycle. On re-induction, developed MRD- Cri
“1”/#7	Azacitidine/0.25 yrs	1	Diploid	*IDH1, NPM1*	None	CR	6 mo	loss of response	Relapsed at the end of 6 consolidation cycles
“1”/#8	7 + 3/0.3 yrs	1	Trisomy11	*NRAS*	None	NR	–	NR	Transitioned to subsequent salvage chemotherapy and bridged to transplant
“1”/#9	CLAD/LDAC/0.3 yrs	1	Diploid	ND	None	NR	–	NR	Transitioned to subsequent salvage chemotherapy
“1”/#10	Azacitidine/refractory	1	Diploid	*TET2,RUNX1, SF3B1*	None	NR	–	NR	Found to have lymphoma.
“1”/#11	DAC, 7+3, DAC-venetoclax/refractory	4	Complex	*TP53*	None	NR	–	NR	Failed other therapies. Transitioned to comfort care

*ASCT, allogeneic stem cell transplant; CIA, clofarabine, idarubicin, and cytarabine; CLAD/LDAC, cladribine, low dose cytarabine; CR, complete remission; Cri, CR with incomplete blood count recovery; DAC, decitabine; FLAG-IDA, fludarabine, idarubicin, cytarabine, G-CSF; FIA, fludarabine, idarubicin, and cytarabine;IA, idarubicin, high dose cytarabine; G- grade; MRD, minimal residual disease; NR, no response; MI, myocardial infarction; mo– months; mo+: in remission and alive; yrs, years; 7 + 3: idarubicin and intermediate dose cytarabine*.

**Figure 2:**
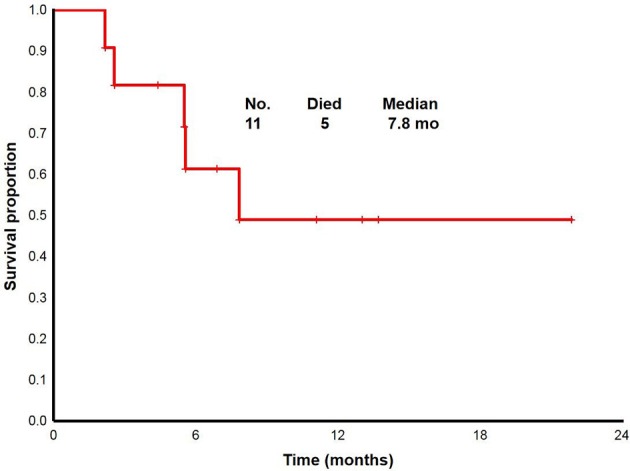
Overall survival for the study population (*n* = 11).

With a median follow up of 6.8 (range, 2.2 to 22+) months, the median overall survival for overall population was 7.8 (range, 2.2 to 22+) months (Figure 2). From 11 patients enrolled: two proceeded to transplant, nine were taken off study either due to no response, progression, or death. One death occurred on study; death occurred while in CRi and was attributable to infectious complications.

Of the 6 patients treated at dose level “0,” there were 2 CRi responders: one of the two patients required a second cycle of induction before achieving response. One patient achieved CR but was positive for minimal residual disease by flow cytometry. Two of responders in this cohort (CR, 1; CRi, 1) were bridged to allogeneic stem cell transplant (ASCT). Of the five patients treated at dose level “1,” one CR was reported. The singular responder continued to receive the next 6 consolidation cycles but subsequently lost response at the end of consolidation treatment.

All responders had received therapy in their first salvage (all three who previously induced with cytarabine-based treatment had CR1 durations lasting > 12 months; fourth responder was previously refractory to azacitidine). Three of four responses were observed in patients with diploid cytogenetics, 1 belonged to favorable risk AML.

### Toxicities

LY2510924 related and unrelated toxicities are outlined in Tables 3, 4, respectively. The most common regimen-related toxicities were diarrhea (54%), nausea/vomiting (45%), mucositis (36%), constipation (27%), and pruritus (27%). Two dose limiting toxicities possibly related to LY2510924 were observed, both developing in the same patient (treated at dose level “0”), them being grade 3 Sweet's syndrome and grade 3 myelosuppression. The Sweet's syndrome lesions resolved with the administration of steroids. No DLTs occurred in the dose level “1” cohort. There were no signs of leukostasis in any of the study patients. However, one patient required to hold the drug due to high leukocyte/high blast count during LY2510924 monotherapy phase.

**Table 3:** LY2510924 related non-hematological toxicities (*n* = 11).

**Toxicity**	**Grade 1–2, *n* (%)**	**Grade 3–4, *n* (%)**	**Total, *n* (%)**
Alopecia	1 (9)		1 (9)
Anorexia	1 (9)	1 (9)	2 (18)
Atrial fibrillation	1 (9)		1 (9)
Bacteremia	1 (9)		1 (9)
Bilirubin elevation	1 (9)		1 (9)
Confusion	1 (9)		1 (9)
Constipation	3 (27)		3 (27)
Dry eyes	1 (9)		1 (9)
Diarrhea	5 (45)	1 (9)	6 (54)
Dizziness	1 (9)		1 (9)
Eye pain	1 (9)		1 (9)
Eye hemorrhage	1 (9)		1 (9)
Fall	1 (9)		1 (9)
Fatigue	1 (9)		1 (9)
Gait disturbance	1 (9)		1 (9)
Gastrointestinal disorders	1 (9)		1 (9)
Headache	1 (9)		1 (9)
Lung infection		1 (9)	1 (9)
Rash, maculopapular	1 (9)	1 (9)	2 (18)
Pruritis		3 (27)	3 (27)
Mucositis	3 (27)	1 (9)	4 (36)
Myelosuppression		1 (9)	1 (9)
Nausea/vomiting	5 (45)		5 (45)
Neuropathy	1 (9)		1 (9)
Oral pain	1 (9)		1 (9)
QTc prolongation	1 (9)		1 (9)
Sweet syndrome	1 (9)		1 (9)
Transaminase elevation	1 (9)		1 (9)
Urinary retention	1 (9)		1 (9)
Total	38	9	47

**Table 4:** LY2510924 unrelated non-hematological toxicities (*n* = 11).

**Toxicity**	**Grade 1–2, *n* (%)**	**Grade 3–5, *n* (%)**	**Total, *n* (%)**
Acidosis		1 (9)	1 (9)
Abdominal pain	2 (18)	1 (9)	3 (27)
Acute kidney injury	1 (9)		1 (9)
Arthralgia/arthritis	3 (27)		3 (27)
Anorexia	2 (18)	1 (9)	3 (27)
Anxiety	2 (18)		2 (18)
Atrial fibrillation		1 (9)	1 (9)
Back pain	1 (9)		1 (9)
Bacteremia		1 (9)	1 (9)
Cellulitis	2 (18)		2 (18)
Colitis		1 (9)	1 (9)
Constipation	2 (18)		2 (18)
Cough	4 (36)		4 (36)
Depression	3 (27)		3 (27)
Diaphoresis	5 (45)		5 (45)
Diarrhea	3 (27)		3 (27)
Dizziness	4 (36)		4 (36)
Dry eyes	2 (18)		2 (18)
Dry mouth	1 (9)		1 (9)
Dyspepsia	1 (9)		1 (9)
Dyspnea	1 (9)		1 (9)
DIC	1 (9)		1 (9)
Ear pain	1 (9)		1 (9)
Edema, pedal	6 (54)		6 (54)
Epistaxis	1 (9)		1 (9)
Erythema multiforme	3 (27)		3 (27)
Eye hemorrhage	1 (9)		1 (9)
Eye infection		1 (9)	1 (9)
Febrile neutropenia		3 (27)	3 (27)
Gastrointestinal disorders	3 (27)		3 (27)
Headache	4 (36)		4 (36)
Hematuria	1 (9)		1 (9)
Herpes simplex	1 (9)		1 (9)
Hyperphosphatemia	1 (9)		1 (9)
Hypertension	2 (18)		2 (18)
Hypokalemia	3 (27)		3 (27)
Hypomagnesemia	1 (9)	1 (9)	2 (18)
Hypophosphatemia		2 (18)	2 (18)
Hypotension	3 (27)		3 (27)
Insomnia	1 (9)		1 (9)
Lung infection	3 (27)	5 (45)	8 (72)
Malaise	4 (36)		4 (36)
Menorrhagia	1 (9)		1 (9)
Mucositis	1 (9)	3 (27)	4 (36)
Muscular atrophy	2 (18)		2 (18)
Myalgia	3 (27)		3 (27)
Nasal congestion	4 (36)		4 (36)
Nausea/vomiting	5 (45)		5 (45)
Palpitations	1 (9)		1 (9)
Paraesthesia	1 (9)		1 (9)
Photophobia	1 (9)		1 (9)
Pneumonitis		1 (9)	1 (9)
Pruritis	1 (9)		1 (9)
Rash, maculopapular	2 (18)	1 (9)	3 (27)
Respiratory failure		1 (9)	1 (9)
Sepsis		4 (36)	4 (36)
Sinusitis	1 (9)		1 (9)
Sore throat	5 (45)		5 (45)
Skin tissue disorders	2 (18)		2 (18)
Small bowel obstruction		1 (9)	1 (9)
Tinnitus	1 (9)		1 (9)
Transaminase elevation		1 (9)	1 (9)
Troponin elevation	1 (9)		1 (9)
Tumor lysis syndrome		1 (9)	1 (9)
Typhlitis		1 (9)	1 (9)
Urticaria	1 (9)		1 (9)
Urinary tract infection	2 (18)		2 (18)
Venous thromboembolism	3 (27)		3 (27)
Total	117	32	149

### Leukemia cell mobilization

**Figure 3:**
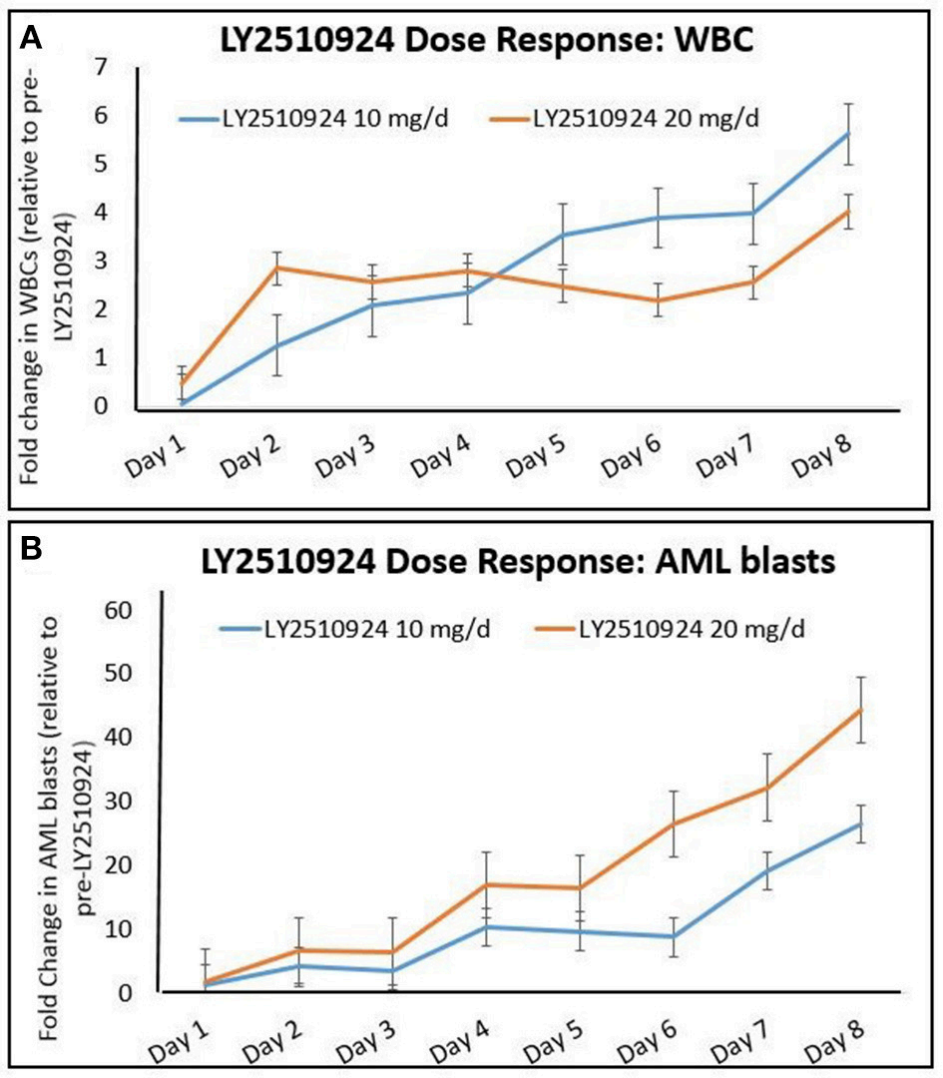
Mobilization of **(A)** total leukocytes (WBC) and **(B)** AML blasts to the peripheral blood over time, after administration of LY2510924 at 10 and 20 mg, from day 1 to day 8. Mean fold changes from the baseline with standard errors are shown.

**Figure 4:**
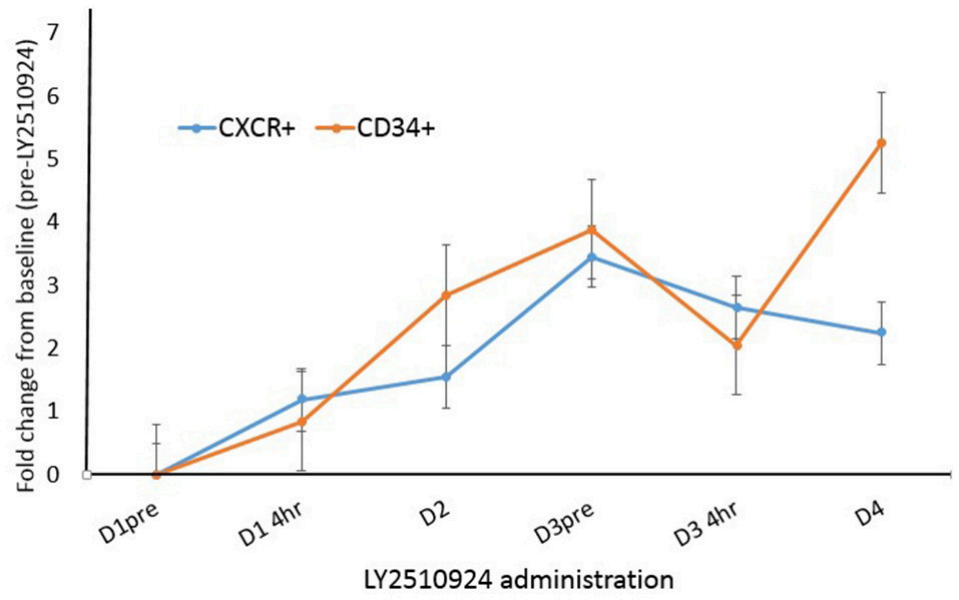
Fold change analysis plotted for CXCR4+ blasts and CD34+ cell populations over time, from day 1 to day 4. Mean fold changes from the baseline with standard errors are shown.

To determine the effect of LY2510924 on leukemic cell mobilization, we analyzed the peripheral blood (PB) samples by complete blood count with differential from 8 separate days: day 1 (baseline before treatment with LY2510924) until day 8 (before treatment with idarubicin/cytarabine). Mean fold changes for the leukocyte and leukemic blast populations (Figures 3A,B, respectively) showed mobilization from the baseline and remained elevated over 7 days of LY2510924 treatment. There was a peak mobilization of leukocytes and PB blasts by days 3 and 4, and they remained elevated at day 8 following administration. Mean fold changes for the CXCR4+ and CD34+ cell populations are plotted in Figure 4. Maximum mean fold changes were 6 for WBCs and > 40 for PB blasts at day 8 (compared to baseline), and 5 for CD34+ cells and 4 for CXCR4+ cells by day 4 (compared to baseline) (Figures 3, 4). There was a mobilization effect even on the CXCR- blast population, although the mean fold changes were not as impressive as observed with the CXCR+ AML blast population. We could not assess the relationship between the dose and leukemic mobilization response due to the sample size of the study population.

**Figure 5:**
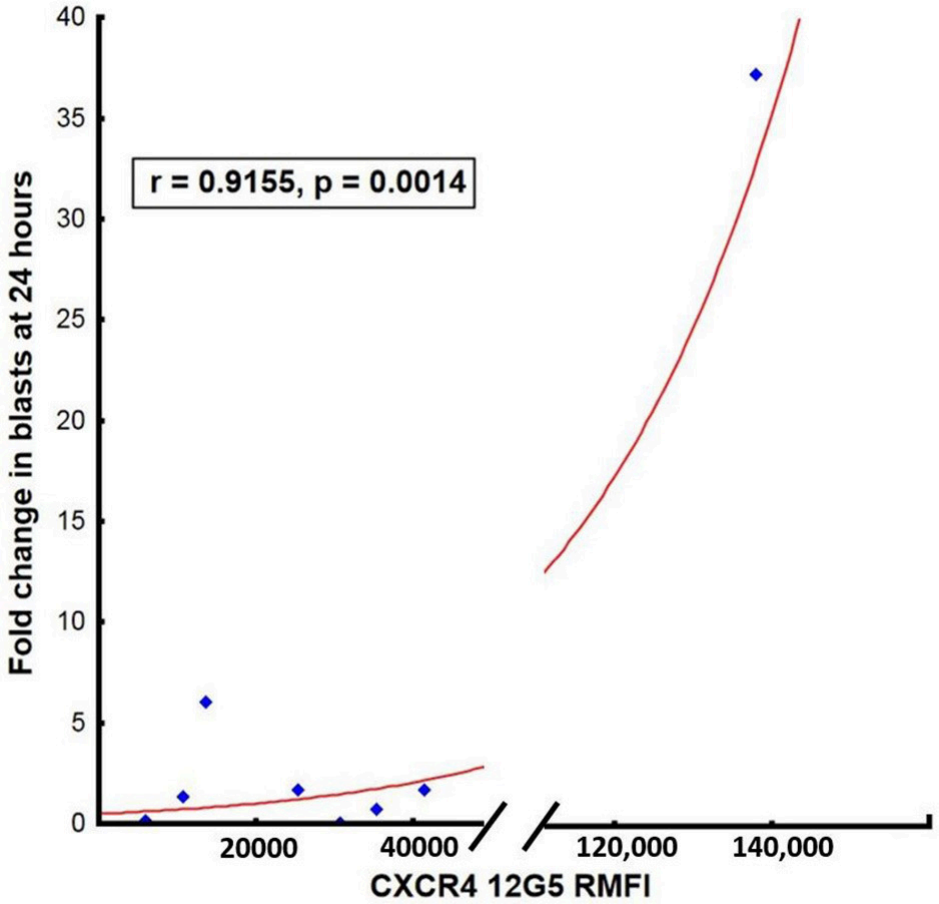
Correlation between surface expression of CXCR4 (MFI measured using clone 12G5) at baseline and mean fold blast change at 24 h post-LY2510924.

The degree of initial leukemic blast mobilization (fold change in leukemic blasts in the peripheral blood between baseline and day 1 post-LY2510924) correlated strongly with the MFI of CXCR4 expression (*r* = 0.98, *p* = 0.001; Figure 5). In contrast to the effect of MFI of the CXCR4 expression, percentage of CXCR4 cells did not have an impact in terms of the median fold change after drug administration. We could not correlate surface expression of CXCR4 molecule to responses to treatment again due to the limited number of clinical responses. On reviewing the day 8 BM samples for a quantitative assessment of leukemic blast numbers, none of the patients had a reduction in BM blast percentage as compared to baseline.

### Expression of CXCR4 and VLA4

**Figure 6:**
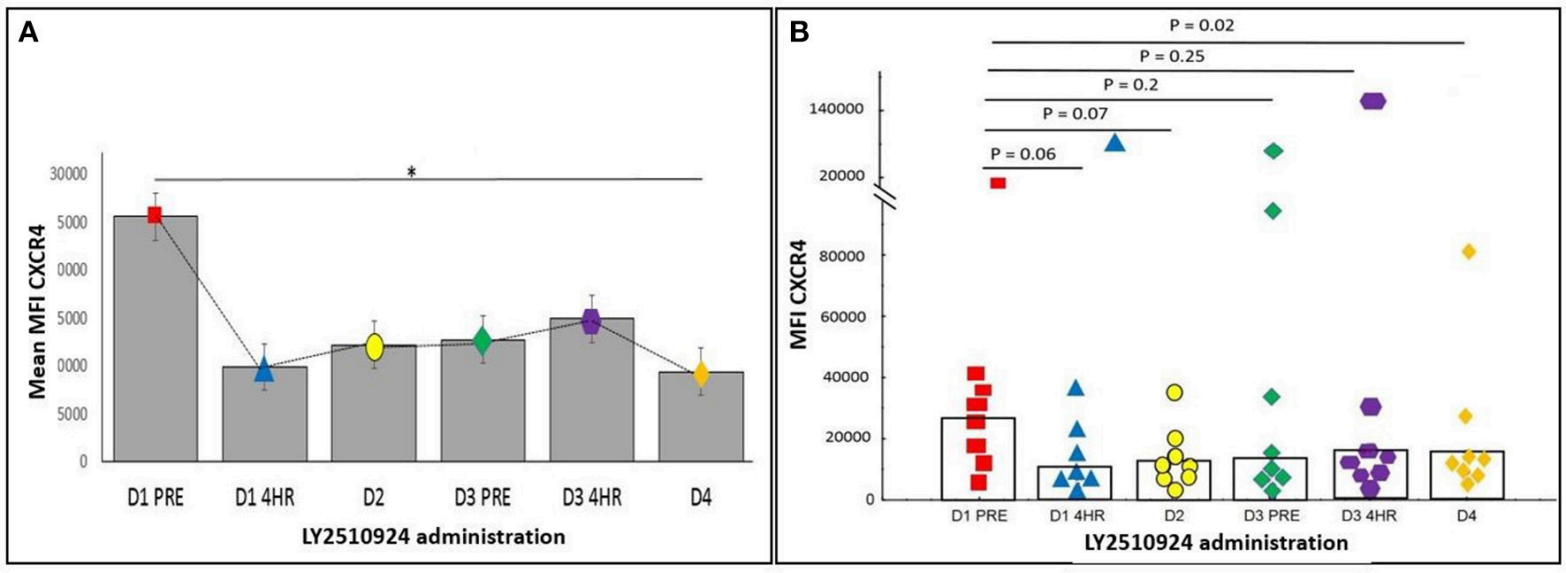
The expression of CXCR4 on peripheral blood AML blasts was determined by flow cytometry using anti-CXCR4 antibody 12G5. **(A)** Mean MFI with standard error over time (*p < 0.05). **(B)** Scatter plot showing each patient's CXCR4 MFI. Values shown are *p*-values comparing baseline with various post-LY2510924 time-points. Statistical comparisons were performed using the Mann-Whitney *U*-test. *p < 0.05.

The expression of CXCR4 and VLA-4 on AML blasts were measured after the administration of LY2510924. We determined the relative mean fluorescence intensity (mean MFI), which corresponds to the surface expression of CXCR4 and VLA-4 on AML blasts. CXCR4 expression and receptor occupancy data (gated on blast cells) was collected in 9 patients, irrespective of their evaluability for clinical outcomes. The expression of CXCR4 on AML blasts was measured using two separate monoclonal antibody (mAb) clones. LY2510924 inhibits the binding of clone 12G5 to CXCR4; reduced antibody binding (expressed as mean MFI) reflects inhibition of CXCR4 receptor occupancy by LY2510924. In contrast, the 1D9 mAb binds to a site on CXCR4 not inhibited by LY2510924 binding, representing true CXCR4 expression on cell surface. We observed a trend toward decrease, albeit statistically non-significantly due to the limited sample size, in 12G5 binding following LY2510924 occurring between pre-treatment and 4 h post-LY2510924 (*p* = 0.08), with binding remaining decreased at day 4 (*p* = 0.02) (Figure 6). In contrast, mean MFI for 1D9 antibody binding was relatively unchanged following treatment with LY2510924 (Supplemental Figure 2). LY2510924 decreased CXCR4 receptor occupancy (% expressing cells) in 6/9 patients: > 70% in two patients (e.g., Supplemental Figure 3), 50% in two patients, and < 50% in three patients; no modulation was observed in 2 patients. VLA4 was highly expressed on AML blasts in all patients and was not modulated by LY2510924 administration (Supplemental Figure 4).

## Discussion

In this article, we demonstrated the safety of LY2510924 with IA combination chemotherapy in AML. Only two dose limiting toxicities (sweets syndrome and myelosuppression) were reported as possibly related to the drug, both in the same patient. This patient had a prolonged myelosuppression with delayed hematopoietic recovery before succumbing to infectious complications. One can only speculate whether the delayed hematopoietic recovery may have been secondary to the loss of normal HSCs that are also mobilized with CXCR-4 antagonists and thereby sensitized to chemotherapy. However, prolonged myelosuppression was not observed in the rest of our study population and neither has it been reported in previous plerixafor/chemotherapy (19, 20, 28) or BL-8040/chemotherapy (30) clinical trials Furthermore, this patient's bone marrow showed trilineage dyspoiesis and the clinical picture was complicated by herpes simplex viremia which was treated with foscarnet, all factors potentially associated with bone marrow suppression. Sweet's syndrome was possibly an on-target toxicity event due to LY2510924 induced mobilization of leukemic cells and subsequent homing to the skin.

One of the concerns with administering LY2510924 in patients with acute leukemia is the occurrence of leukostasis leading to vascular or pulmonary complications (33). This is due to the mobilizing effect of blasts into the peripheral circulation (34). While there was no symptomatic leukostasis in any of the study participants, one patient required to hold LY2510924 due to high leukocyte/blast counts arising during the LY2510924 administration. Close monitoring of counts is therefore emphasized in every patient during its administration in the monotherapy phase.

The ORR of 36% of the combination of LY2510924 and IA chemotherapy in our study population was comparable with response rates of 30–38%, in a study published from our institution, with IA based chemotherapy alone (35). It must be noted however that response rates with intensive chemotherapy in the R/R setting vary widely from 4 to 80% depending on several characteristics including age, cytogenetics, CR1 duration, number of prior salvage chemotherapy regimens, and the specific treatment regimen used (35–53). Pertinently, all our study responders were in their first salvage of treatment, harbored non-adverse cytogenetics, with CR1 durations lasting more than a year amongst those previously induced with cytarabine based chemotherapy. Our interpretation of the effect of treatment on disease free survival and OS was limited by the small sample size estimates and short follow-up time. Nevertheless, the survival data in our study compared favorably to historical OS estimates of 5.9 and 4.7 months in first- and second- salvage AML, respectively (54, 55). In this context, survival in R/R AML is highly influenced by the post-remission therapy, ASCT being the only consistently curative option with patients rarely achieving durable remissions with non-transplant strategies (56). While two of the study responders were successfully bridged to ASCT, one continued post-remission chemotherapy and relapsed by the end of consolidation treatment.

In a phase 1/2 study of plerixafor (a 1st generation CXCR-antagonist) in combination with MEC in relapsed/refractory AML, treatment with plerixafor demonstrated only a 2-fold mobilization of leukemic blasts into the peripheral blood, with a return to baseline within 12 h (28). BL-8040 is a far more robust stem cell mobilizer (57) than plerixafor and early phase clinical trial data support its efficacy in AML (30). In a phase 1/2 trial of patients with R/R AML (NCT01838395) (30), 2 days of BL-8040 treatment was associated with a 40-fold increase in immature AML progenitors from the marrow. While LY2510924 lacks the pro-apoptotic properties of BL-8040 (58), a > 40-fold increase in AML progenitors from the marrow was similarly noted in our study. Also, a sustained CXCR-blockade (up to 24 h post-dosing) as confirmed by the FACS analysis. Thus, LY2510924 shares the superior pharmacodynamic and blast cell mobilization properties of BL-8040, which has implications for sensitization to chemotherapy. We were unable to associate treatment response with CXCR4 inhibition due to the limited numbers of patients analyzed.

Similar to the case with plerixafor (28, 59), LY2510924 was shown to inhibit receptor internalization causing minimal compensatory upregulation of surface CXCR4 expression in a previous report (60). Increased surface expression is associated with enhanced CXCR4 function and may mitigate the intended anti-apoptotic effect with CXCR4 blockade (28, 61). LY2510924 related CXCR4 blockade was not associated with an upregulated surface CXCR4 expression (measuring using 1D9 mAb) in our study.

Two studies, a phase I study of LY2510924 and durvalumab in patients with RCC and a phase II study of LY2510924 and carboplatin/etoposide in SCLC, have yielded disappointing results in terms of efficacy (20, 62). The dose of LY2510924 employed in both studies was 20 mg, based on data from a phase I trial (19). Pharmacodynamic monitoring in our study population showed incomplete target inhibition irrespective of dose (i.e., 10 or 20 mg), the majority achieving < 50% inhibition of receptor occupancy. Recent safety data from the phase 1a study (NCT02737072) in solid tumor patients showed no dose limiting toxicities in any of the 3 cohorts with daily dosing of LY2510924 at doses of 20, 30, or 40 mg and the combination of durvalumab. Upon discussion with the sponsor, exploration of a higher (30 mg) dose of LY2510924 in combination with idarubicin/cytarabine chemotherapy was felt warranted, in an additional escalation cohort prior to expansion.

In conclusion, combining LY2510924 with IA chemotherapy is safe in relapsed patients with AML. One of the major limitations of our study is the low patient sample size precluding an in-depth evaluation of biologic correlates of treatment response. However, the findings in this study suggest that the drug holds therapeutic promise and has a superior pharmacologic profile over plerixafor. We emphasize count monitoring in every patient with AML who receives this agent. LY2510924 at dose of 10 and 20 mg/day suppresses CXCR4 receptor occupancy in some but not all patients, and a higher dose of LY2510924 is needed for complete CXCR4 receptor occupancy. The FDA has approved the higher dose of 30 mg dose and protocol amendment is approved (NCT02652871).

## Author contributions

PraB and MaK involved in data collection, statistical analysis, writing the manuscript. GB, KN, WW, PriB, EJ, ZE, JB, YA, JC, HK, and MA involved in providing patients for the study, writing and reviewing the manuscript. XH was the statistical collaborator for the trial, and involved in providing statistical input for the manuscript. MyK involved in designing the study, reviewing the manuscript. AD and AP involved in organizing patients and data for the study, collecting and managing data. AC and LH involved in performing pre-clinical studies and gathering pre-clinical data and reviewing the manuscript. Mak involved in designing the study, writing and reviewing the manuscript.

### Conflict of interest statement

The authors declare that the research was conducted in the absence of any commercial or financial relationships that could be construed as a potential conflict of interest.
