# Supplementary material for: Initial Report of a Phase I Study of LY2510924, Idarubicin, and Cytarabine in Relapsed/Refractory Acute Myeloid Leukemia
Source: Front Oncol. 2018 Sep 24;8:369. doi: 10.3389/fonc.2018.00369 (PMC6167965; doi:10.3389/fonc.2018.00369)
Supplement: Supplementary file 1 [file Table_1.DOCX]

**Supplemental figure 1:** Gating strategy for AML stem cells (CD34+ CD38- CD123+ blast phenotype)

**
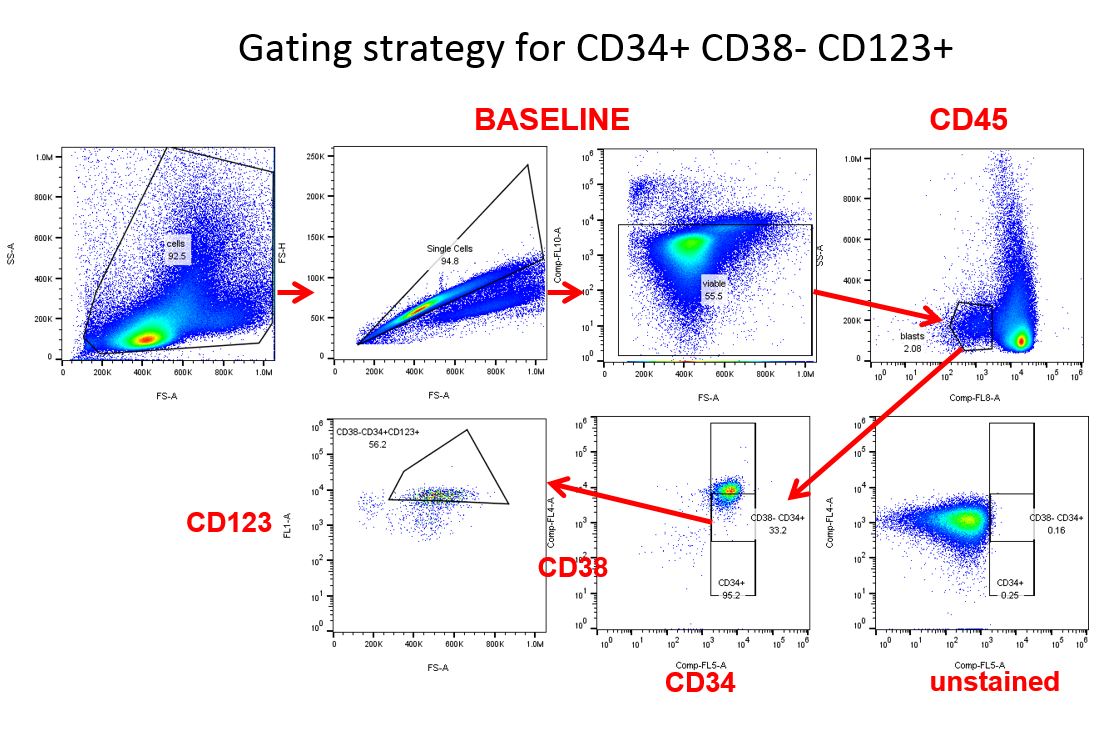
**

**Supplemental figure 2:** VLA4 and CXCR4 expressing (CD45 dim (blasts) + monocytes + CD123+) cells as a function of time in patient # 1. Percentage of cells expressing VLA4 increases with time peaking at D3 (pre- and 4hour). While there is no modulation in CXCR4 expression measured by clone 1D9, CXCR4 surface expressing cells (clone 12G5) decrease over time, with a nadir at D2.

**
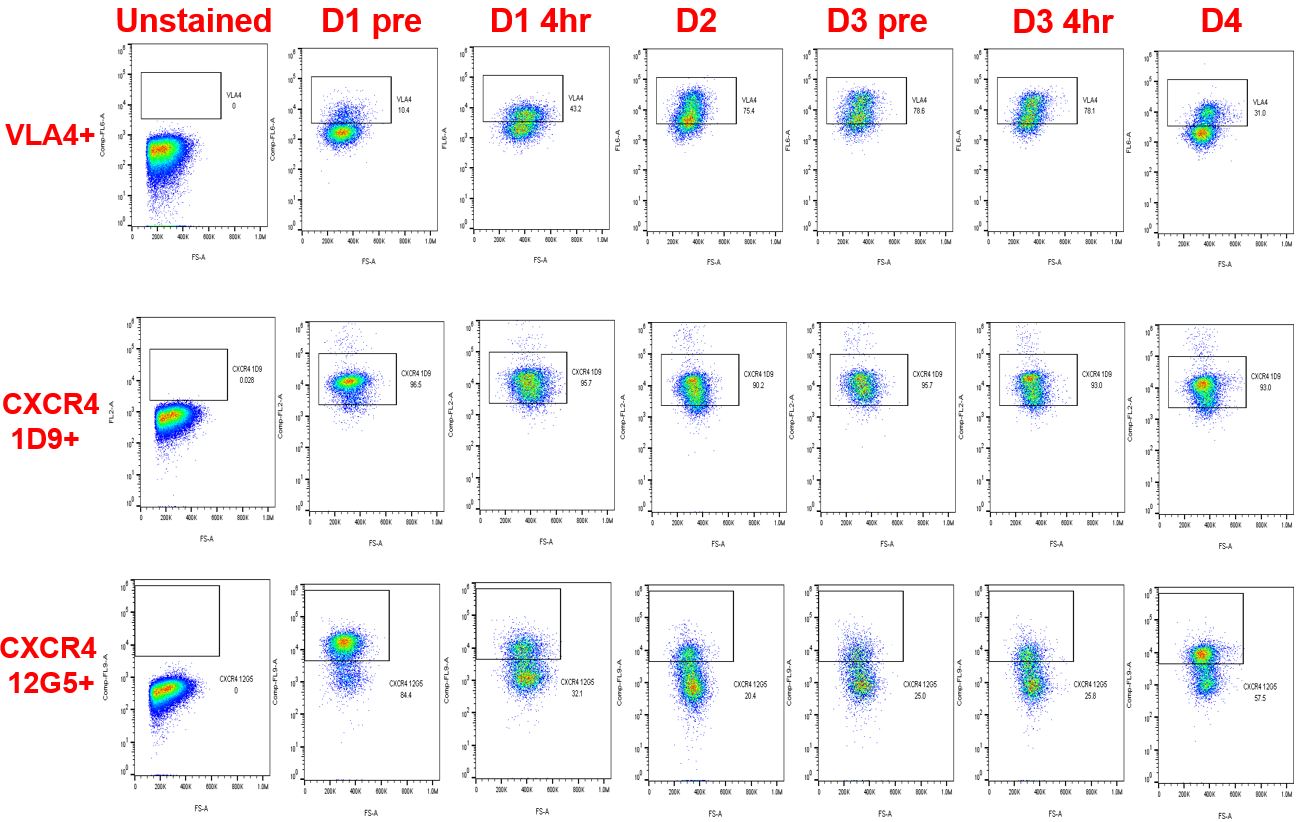
**

**Supplemental figure 3:** Expression of CXCR4 on AML blasts as a function of time. The expression of CXCR4 on peripheral blood AML blasts was determined by flow cytometry using anti-CXCR4 antibody 1D9. The mean MFI with SE are shown

**
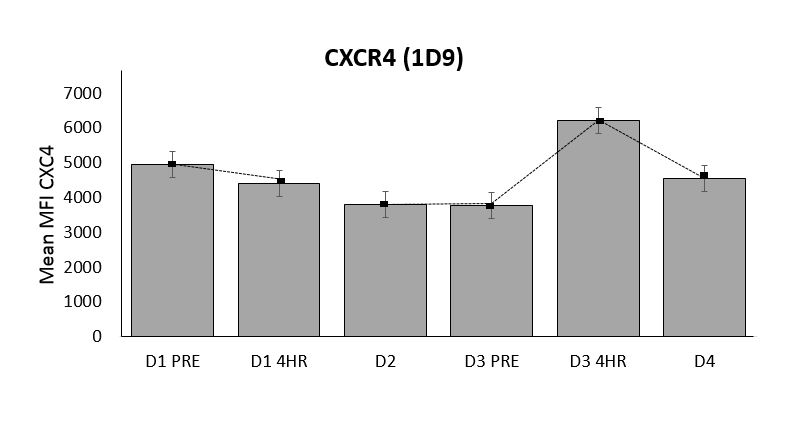
**

**Supplemental figure 4:** Expression of VLA4 on AML blasts as a function of time. The mean MFI with SE are shown

**
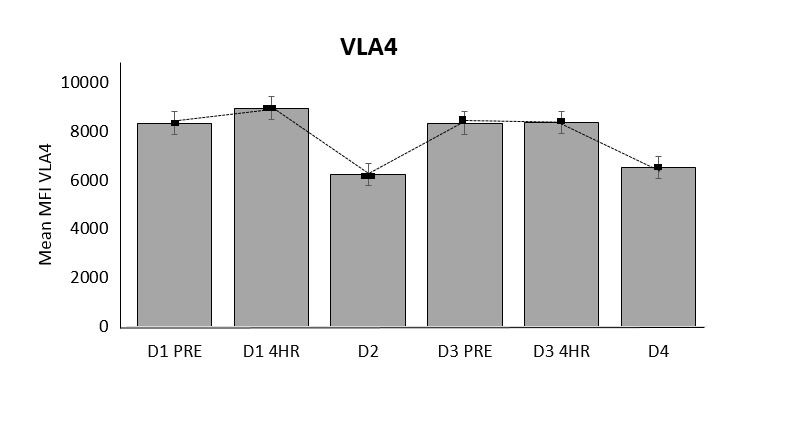
**
